# The Light Chain Defines the Duration of Action of Botulinum Toxin Serotype A Subtypes

**DOI:** 10.1128/mBio.00089-18

**Published:** 2018-03-27

**Authors:** Sabine Pellett, Marite Bradshaw, William H. Tepp, Christina L. Pier, Regina C. M. Whitemarsh, Chen Chen, Joseph T. Barbieri, Eric A. Johnson

**Affiliations:** a Department of Bacteriology, University of Wisconsin, Madison, Wisconsin, USA; b Department of Microbiology and Molecular Genetics, Medical College of Wisconsin, Milwaukee, Wisconsin, USA; University of Pittsburgh School of Medicine

**Keywords:** BoNT, botulinum neurotoxin, duration of action, hybrid toxin, subtype

## Abstract

Botulinum neurotoxin (BoNT) is the causative agent of botulism and a widely used pharmaceutical to treat a variety of neurological diseases. BoNTs are 150-kDa protein toxins organized into heavy chain (HC) and light chain (LC) domains linked by a disulfide bond. The HC selectively binds to neurons and aids cell entry of the enzymatically active LC. There are seven immunological BoNT serotypes (A to G); each serotype includes genetic variants, termed subtypes. Only two subtypes, BoNT/A1 and BoNT/B1, are currently used as therapeutics. BoNT serotype A (BoNT/A) subtypes A2 to A8 show distinct potency, duration of action, and pathology relative to BoNT/A1. Specifically, BoNT/A3 possesses shorter duration of action and elicits distinct symptoms in mice at high toxin doses. In this report, we analyzed the roles of LC and HC of BoNT/A3 for duration of action, neuronal cell entry, and mouse pathology by using clostridium-derived recombinant hybrid BoNTs consisting of reciprocal LC and HC (BoNTA1/A3 and BoNTA3/A1). Hybrid toxins were processed in their expression host to a dichain BoNT consisting of LC and HC linked via a disulfide bond. The LC and HC defined BoNT potency in mice and BoNT toxicity for cultured neuronal cells, while the LC defined the duration of BoNT action in cell and mouse models. Protein alignment identified a previously unrecognized region within the LC subtype A3 (LC/A3) relative to the other LC serotype A (LC/A) subtypes (low primary acid homology [LPH]) that correlated to intracellular LC localization. This study shows the utility of recombinant hybrid BoNTs with new therapeutic potential, while remaining sensitive to antitoxins and therapies to native BoNT.

## IMPORTANCE

Botulinum neurotoxins are the most potent protein toxins for humans and potential bioterrorism threats, but they are also widely used as pharmaceuticals. Within the large family of BoNTs, only two subtypes are currently used as pharmaceuticals, with a large number of BoNT subtypes remaining as untapped potential sources for unique pharmaceuticals. Here, two recombinant hybrid toxins were engineered, consisting of domains from two BoNT subtypes that possess distinct duration of action and activity in human neurons and mice. We define the functional domains responsible for BoNT action and demonstrate creation of functional hybrid BoNTs with new therapeutic potential, while remaining sensitive to antitoxins and therapies to native BoNT.

## INTRODUCTION

Botulinum neurotoxins (BoNTs) are a large and diverse family of potent neurotoxins produced by a heterogeneous group of Gram-positive bacteria, primarily *Clostridium botulinum*. In a susceptible host, BoNT causes flaccid neurological paralysis by blocking muscle innervation at the neuromuscular junction. BoNTs are AB type protein toxins, consisting of a heavy chain (HC), which efficiently binds neurons and facilitates light chain (LC) entry into the cell cytosol. LCs are zinc-dependent endopeptidases, which specifically cleave neuronal SNARE (soluble *N*-ethylmaleimide-sensitive factor attachment protein receptor) proteins. This multistep intoxication process underlies BoNT neuronal specificity and potency. Seven immunologically distinct BoNT serotypes (A to G) comprise more than 100 genetic variants and several chimeric toxins that encode LC of one serotype fused to the HC of another serotype or subtype (1, 2).

BoNT intoxication yields long-lasting paralysis (weeks to months) at the neuromuscular junction, with durations of action unique to each serotype. BoNT serotype A (BoNT/A) subtypes have the longest known duration, with paralysis lasting from several months to ~1 year depending on the dose of the toxin. For this reason, BoNT serotype A subtype A1 (BoNT/A1) is the BoNT type primarily used for pharmaceutical purposes to achieve long-lasting (3 to 6 months) therapeutic flaccid paralysis with local injection. Interestingly, recent analyses of several BoNT/A subtypes showed that the duration of action can vary among the subtypes within one serotype (3–5). Like BoNT/A1, BoNT/A2, BoNT/A4, BoNT/A5, and BoNT/A6 have long durations of action, but BoNT/A3 has significantly shortened duration of action in both cultured neurons and a mouse model of botulism (3, 4).

Duration of action of BoNTs is a key characteristic affecting both their utility as pharmaceuticals and their severity of toxicity. However, little is known about the molecular mechanisms underlying duration of action. BoNT/E1 has a short duration of action (2 to 4 weeks) relative to BoNT/A1. Expression in N18 neuroblastoma cells indicate that the LC/E1-YFP fusion protein (LC of serotype E subtype E1 [LC/E1] fused to the yellow fluorescent protein [YFP]) is degraded by the ubiquitin-proteasome system, while LC/A1-YFP was stable (6). Recent evidence implicates a deubiquitinase in the removal of ubiquitin from the BoNT/A1 LC inside neuronal cells as a potential molecular mechanism underlying the long duration and apparent resistance of LC/A1 to ubiquitin-mediated degradation (7). Other studies reported transiently expressed LC/A1-GFP (green fluorescent protein) in PC12, Neuro-2A, and nonneuronal cells localized in a punctate manner to discrete areas of the plasma membrane, while the LC/E1-GFP localized to the cell cytosol with nuclear exclusion (8–10). Mutations that reduced LC/A1 membrane localization reduced SNAP-25 cleavage in Neuro-2A cells (10). However, studies in N18 neuroblastoma cells reported plasma membrane distribution and colocalization of LC/A1-RFP (red fluorescent protein) and LC/E1-YFP (6). Complicating the interpretation of these observations, several studies in mice suggest extensive neuron remodeling and neuritogenesis with formation of new and functional synapses after BoNT/A1 intoxication of a neuromuscular junction (NMJ) (6, 11–14). An extensive study in mice using elegant techniques of *in vivo* imaging and electrophysiological measurements of a single NMJ after BoNT/A1 injection showed that neurite sprouting occurs in NMJs after BoNT/A1 intoxication (15, 16). These data indicate that in mice, formation of functional but transient new synapses can substitute for the intoxicated synapse until the original intoxicated synapse has recovered.

BoNT intoxicates neuronal cells by binding to dual host receptors, a ganglioside and protein receptor, on the neuronal cell surface, followed by endocytosis and translocation of the LC into the cell cytosol (17–21). In the cell cytosol, the disulfide bond connecting LC and HC is reduced, and LC persists and remains active inside the cell cytosol (4, 22–24). While LC is assumed to be solely responsible for the duration of BoNT action, little direct evidence supports this hypothesis. In addition, the fate of the HC after cell entry and disulfide bond cleavage is unknown.

To address the basis for BoNT longevity directly, we engineered hybrid toxins consisting of LC-HC chimeras of the long-lived BoNT/A1 and short-lived BoNT/A3 produced in a native expression host. In several experimental systems, the LC appeared to be solely responsible for the duration of BoNT action, while LC and HC both contributed to the onset and potency of BoNT action. In addition, we show distinct localization of the LC/A1 and LC/A3 in cultured neuronal cells. Thus, our studies show that recombinant intraserotype hybrid BoNTs can be engineered with potentially new therapeutic characteristics, while remaining sensitive to antitoxins and therapies to native BoNT.

## RESULTS

### BoNT/A3 has a unique molecular organization.

**FIG 1:**
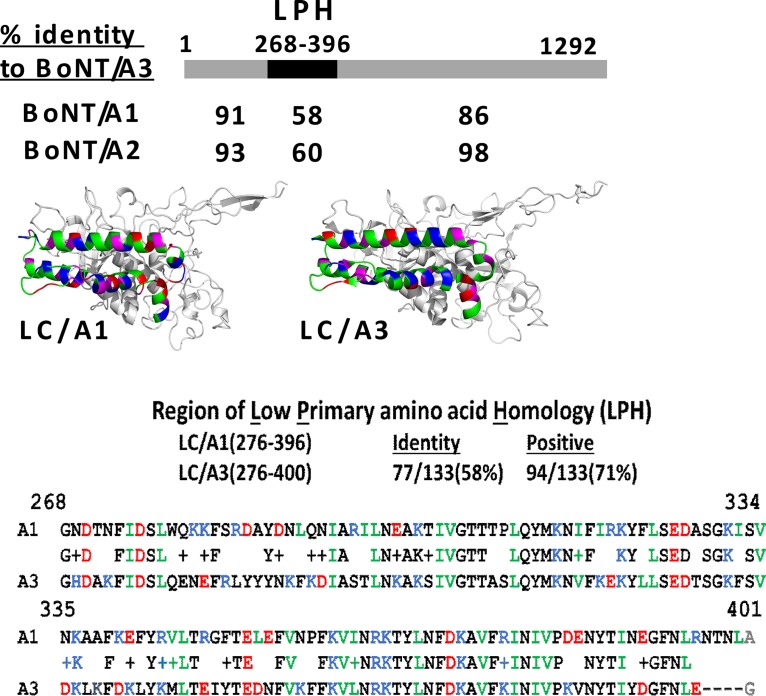
BoNT/A3 alignment and model with BoNT/A1 and BoNT/A2. (Top) BoNT/A3 schematic. BLASTP alignments showed the N-terminal 267 amino acids of BoNT/A3 had 91 and 93% homology, residues 268 to 393 had 58 and 60% homology, and residues 395 to 1292 had 86 and 98% homology with BoNT/A1 and BoNT/A2, respectively. (Middle) LC/A3 modeled on LC/A1. LC/A3(268-393) was modeled on the respective region of LC/A1 (PDB accession number 3BTA). Acidic (red), basic (blue), and aliphatic (green) amino acids are indicated. (Bottom) This region of low primary amino acid homology was termed the LPH. Alignment of LC/A1(268-398) and LC/A3(268-393) are shown. Note the 4-amino-acid deletion at the C terminus of the LPH, which is only common to the BoNT/A3 among the BoNT/A subtypes.

The primary amino acid sequences of three previously described BoNT/A3 isolates are identical, while a fourth isolate and that of strain Loch Maree differs by only 2 amino acids (25, 26), all encoding 1,292 amino acids. Alignment assessment, using the National Library of Medicine BLASTP resource (27), showed the N-terminal 276 amino acids of BoNT/A-LC/A3(1-266) are 91 and 93% identical to BoNT/A1 and BoNT/A2, respectively, whereas amino acids (aa) 267 to 396 of BoNT/A-LC/A3 are only 60 and 64% identical with the respective regions of BoNT/A1 and BoNT/A2, respectively. The amino acid region from amino acids 267 to 396 of low amino acid homology between A3 and A1 or A2 will be termed the LPH (low primary amino acid homology) region. The respective LPH regions of LC/A1 and LC/A2 share 90% homology. BoNT/A3 aa 397 to 1292 is 86 and 98% identical with the respective regions of BoNT/A1 and BoNT/A2 (Fig. 1). In addition, BoNT/A3 possesses a 3-amino-acid deletion starting at amino acid 397 relative to the other BoNT/A subtypes. Overall, BoNT/A3 appears to be derived from a recombination event within BoNT/A2, resulting in a hybrid toxin consisting of a unique LC/A3 and HC/A2. Amino acid alignment and structural modeling of LC/A3 on LC/A1 (PDB accession number 3BTA) showed that the overall structural organization of the LPH was conserved between LC/A3 and LC/A1 (Fig. 1). Despite the lower primary amino acid homology, the LPH region of LC/A3 contained each of the amino acids (S pocket residues) implicated in LC catalysis (28). However, the LPH of LC/A3 contained a cluster of basic amino acids that are absent in LC/A1. The high level of BoNT/A3 and BoNT/A2 HC homology suggests that BoNT/A3 isolates are a BoNT/A3-A2 subtype. The source of the A3 LPH may be due to a recombination event between a yet undefined BoNT/A subtype and BoNT/A2 or an unusually high number of accepted amino acid substitutions within the LPH of BoNT/A2 that eventually yielded the LC/A3-LPH.

### BoNT/A subtypes and chimeras produced in a clostridial expression system.

**FIG 2:**
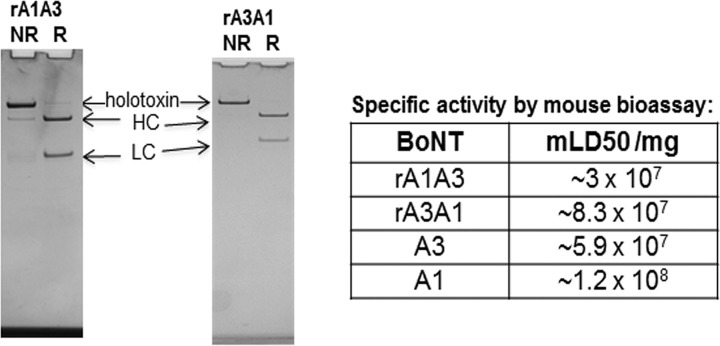
SDS-PAGE gels of purified 150-kDa chimeric BoNT/A subtype toxins and specific activity in mice. The chimeric BoNTs were expressed in a clostridial expression system and purified by biochemical methods. rA1A3, recombinant A1A3. (Left) Nonreduced (NR) and reduced (R) samples were analyzed by SDS-PAGE gels analysis. (Right) Specific toxicity was determined in mice and compared to that of the parent BoNT/A subtypes. mLD50, mouse 50% lethal dose.

Previous studies indicated several biological and functional differences between BoNT/A1 and BoNT/A3, including a shorter duration of action of BoNT/A3, differential potency in human cell models, and distinct symptoms in mice injected with high doses of the toxins (3, 4, 29). In order to determine the roles of LC and HC in these functions, hybrid toxins comprising BoNT/A1A3 and BoNT/A3A1 (BoNT/LCHC) were constructed. To limit the influences of added structural elements, no epitope tags or linkers were included, such that the hybrid toxins contained only the LC and HC joined by the native disulfide bond. The constructs were expressed in an expression host derived from *C. botulinum* strain Hall A-hyper to enable proper posttranslational processing. Analysis of the purified toxins showed a 150-kDa protein that was processed to the dichain form as evidenced by reduction to the HC and LC (Fig. 2).

### HC primarily contributes to BoNT/A potency in mice.

In intraperitoneal mouse bioassays, the specific activity of hybrid toxin BoNT/A1A3 was similar to the specific activity of BoNT/A3 and about threefold lower than that of BoNT/A3A1. The specific activity of BoNT/A3A1 was only slightly reduced relative to that of BoNT/A1. These data indicate that the hybrid toxins were functional and had proper posttranslational processing in the native clostridial expression host. Overall, the specific toxicities of BoNT/A1A3 and BoNT/A3 are approximately two- to threefold lower than those of BoNT/A1 and BoNT/A3A1. Thus, HC function (receptor interactions, entry, and/or LC translocation), not LC function (catalysis or duration of toxin action), is the rate-limiting step in defining the specific activity of BoNT/A1 and BoNT/A3 in the mouse model of botulism.

Previously we observed a difference in symptoms of mice injected with high doses of BoNT/A1 and BoNT/A3, where mice injected with BoNT/A1 displayed typical botulism symptoms of ruffled fur, wasped waist, slowly increasing paralysis, and spasticity before death, whereas mice injected with BoNT/A3 showed increasing paralysis of the limbs and minimal spasticity before death (29, 30). Differences in symptoms in mice injected with chimeric toxins were subtle, with not every mouse fitting an exact symptom profile. There was a trend for animals injected with BoNT/A1A3 to display symptoms similar to mice injected with BoNT/A1, and mice injected with BoNT/A3A1 to display symptoms similar to mice injected with BoNT/A3. The main differences were that all five mice injected with either BoNT/A1 or BoNT/A1A3 showed strong spasticity prior to death, whereas only two mice injected with BoNT/A3 or three mice injected with BoNT/A3A1 showed strong spasticity and the other mice showed much milder spasticity. In addition, mice injected with BoNT/A3 or BoNT/A3A1 appeared to suffer from more pronounced walking impairment due to apparent paralysis of all four legs. Since neither hybrid recapitulated the parent, LC and HC functions contribute to the botulism symptoms, with LC potentially more influential than HC functions.

### LC and HC contribute to BoNT/A3 potency in cultured human neurons.

**FIG 3:**
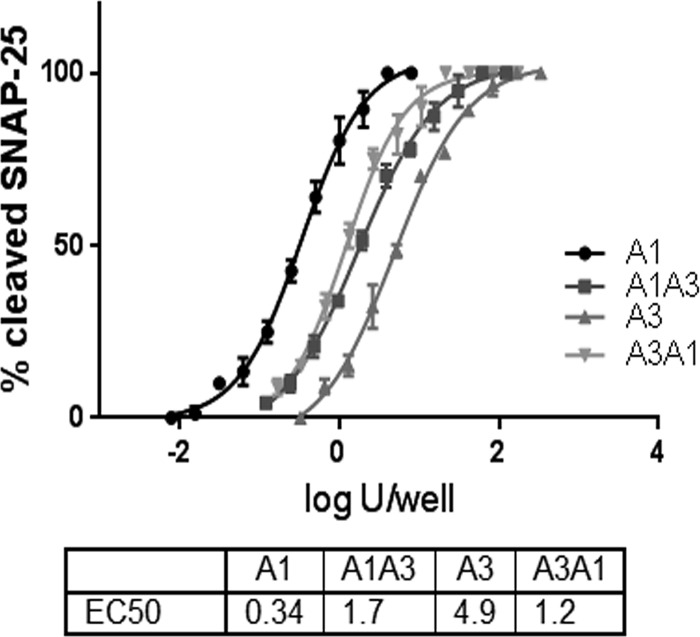
Activity of BoNT/A subtype hybrids in human neurons. Human iPSC-derived neurons were exposed to the indicated serial dilutions of BoNT/A1, BoNT/A3, BoNT/A1A3, and BoNT/A3A1 for 48 h, and cell lysates were analyzed for SNAP-25 cleavage by Western blotting. Data from three Western blots were quantified by densitometry and data plots, and EC_50_ values were generated using Prism6 software. EC_50 _values were determined from nonlinear regression curve fits (four parameters). The same biological activities of the toxins were compared. Log U/well on the *x* axis is the logarithm of EC_50_ or the amount required to kill 50% of mice within 4 days after intraperitoneal injection. *n* = 3. U, LD_50_ units.

Previous analyses of BoNT/A1 and BoNT/A3 indicated that cultured human neurons were more than 50-fold less sensitive to BoNT/A3 than to BoNT/A1. To determine whether the lower sensitivity of human neurons to BoNT/A3 was due to LC or HC function, the hybrid toxins were analyzed in the same human neuronal cell model. Human induced pluripotent stem cell (hiPSC)-derived neurons (iCell neurons) were exposed to serial dilutions of the hybrid toxins and the parental toxins in parallel. BoNT/A1 had an activity similar to that found in previous studies (about 0.3 50% lethal dose [LD_50 _] unit/well), while BoNT/A3 activity was about twofold higher than previously reported (about 5 LD_50_ units/well), yielding a differential sensitivity between BoNT/A1 and BoNT/A3 of ~15-fold. We attribute the change in relative potency of BoNT/A3 to stock-specific differences. The sensitivity of cells to both hybrid toxins were between 1 and 2 LD_50_ units/well, between the activity of the parental toxins (Fig. 3). Thus, LC and HC functions contribute to the lower BoNT/A3 potency in cultured human neurons.

### LC defines BoNT duration of action.

**FIG 4:**
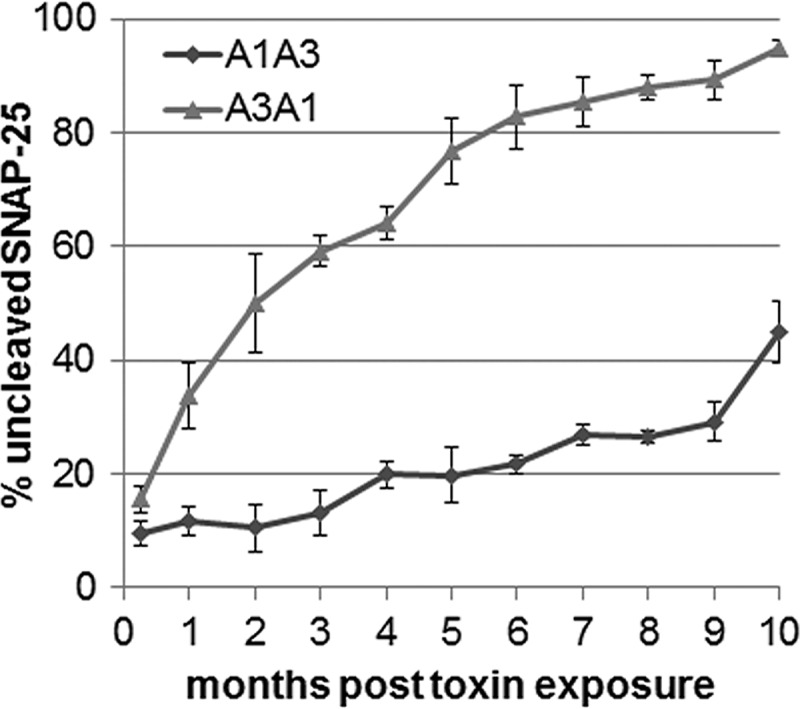
Duration of action of BoNT/A1A3 and A3A1 subtype hybrids in cultured neurons. Cultured primary rat spinal cord cells were exposed to similar concentrations of the toxins for 48 h, followed by removal of all extracellular toxin and thorough washing of the cells. Cells were then incubated in fresh medium for up to 10 months. At least triplicate samples of the cells were harvested monthly, and SNAP-25 cleavage was determined by Western blotting and densitometry. The percentage of uncleaved SNAP-25 is shown in the graph, indicating recovery from the respective toxins’ LC catalytic activity.

Previous data indicated that the duration of action of BoNT/A3 in cultured neurons and in mice was significantly shorter than those of BoNT/A1. Duration of hybrid toxin action in cultured primary rat spinal cord neurons was determined as previously described (4) by first exposing neuronal cell cultures to the same amount of toxin, followed by removal of the toxin and continued incubation and periodically determining SNAP-25 cleavage in cell samples over the next 10 months. BoNT/A1A3 resulted in limited gradual recovery in full-length SNAP-25, similar to previous observations for BoNT/A1 (4), while the BoNT/A3A1 hybrid resulted in an earlier, steady recovery that was nearly complete after 5 months, similar to that of BoNT/A3 (4) (Fig. 4). Thus, LC function defines the intracellular persistence of BoNT action in primary neuronal cell cultures.

**FIG 5:**
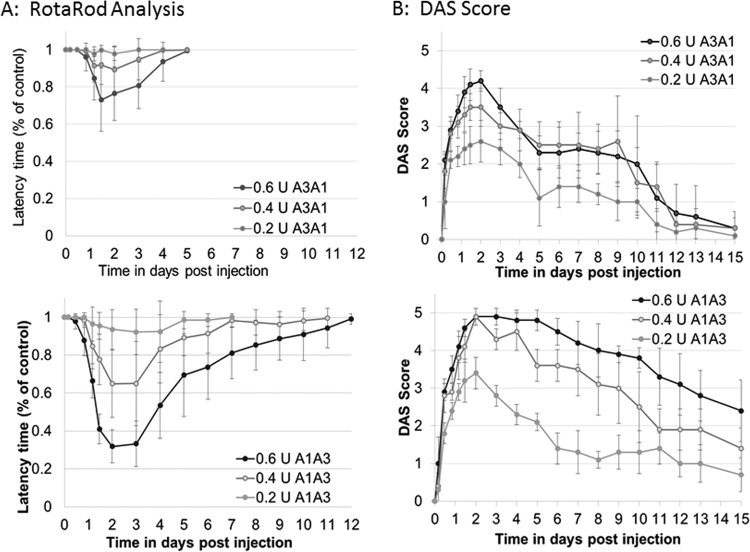
Duration of action of BoNT/A1A3 and BoNT/A3A1 after local injection in mice. Groups of mice (five mice in each group) were injected locally into the gastrocnemius muscle with the indicated doses of BoNT/A1A3 or BoNT/A3A1 diluted in GelPhos buffer. U, LD_50_ units. (A) Duration of motor neuron deficiency was quantitatively measured by rotarod analysis using an accelerating rod (4 to 40 rpm over 5 min) and is shown as latency on a scale of 0 to 1 with 0 being maximum latency and 1 being no measured latency. (B) Duration of local paralysis symptoms was quantitatively assessed by DAS (digital abduction score) analysis on scale from 1 to 5. The average ± standard deviation (error bar) for each group (with five mice in each group) are shown at the indicated time points.

Functional recovery from the hybrid toxins was further examined in the mouse model as previously done for BoNT/A1 and BoNT/A3 (3). After local injection of BoNT/A1A3 and BoNT/A3A1 into the gastrocnemius muscle of the right hind limb, local paralysis was assessed by the digital abduction score (DAS), and overall motor neuron deficiency of the animals was measured by rotarod analysis. Mice injected with BoNT/A1A3 exhibited dose-dependent overall motor neuron deficiency similar to that previously observed for mice injected with BoNT/A1, as evidenced by their inability to remain running on the accelerating rotarod. Full recovery from motor neuron deficiency at the highest dose of 0.6 LD_50_ units required about 12 days, which is similar to recovery previously observed with BoNT/A1 at a similar dose (3). Similarly, mice injected with BoNT/A1A3 showed a dose-dependent local paralysis as evidenced by an increase in the DAS, with maximum paralysis reached around day 2, followed by a slow decrease in the DAS over the next 13 days. Recovery from local paralysis was not complete after 15 days and followed a pattern similar to that previously observed with BoNT/A1 (3). However, mice injected with the same doses of BoNT/A3A1 exhibited only mild overall motor neuron deficiency on the rotarod with recovery at the highest dose complete by day 5, which is similar to previous observations for BoNT/A3 (3). The onset of local paralysis measured by DAS was similar to that in mice injected with BoNT/A1A3, but paralysis was slightly less severe, reaching a maximum score of about 4.2 at the highest dose compared to a maximum score of 4.9. Recovery was significantly faster and nearly complete after 12 days, similar as previously observed with BoNT/A3 (Fig. 5). These data demonstrate that the shorter duration of action of BoNT/A3 is solely determined by the action of LC.

### LC/A1 and LC/A3 have unique intracellular localization.

**FIG 6:**
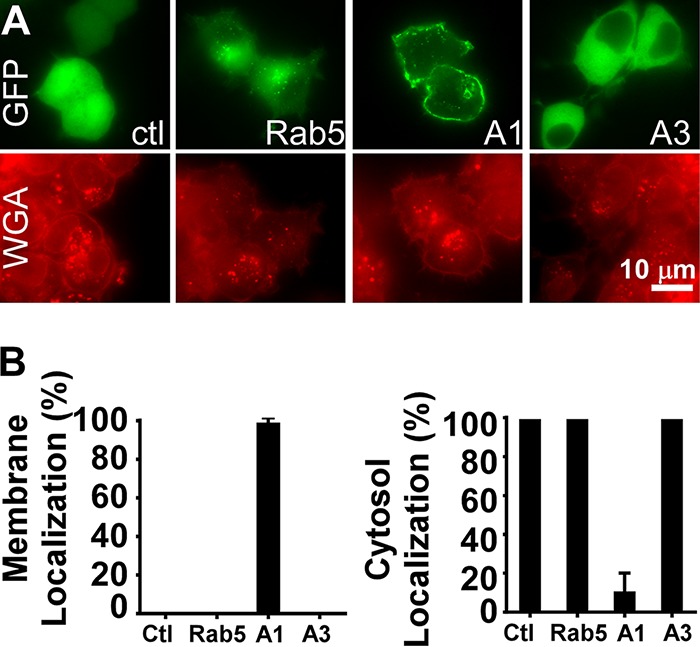
Intracellular localization of BoNT LC/A subtypes. pEGFP-C3-derived plasmids expressing EGFP-LC/A1 (A1), EGFP-LC/A3 (A3), EGFP-Rab5 (Rab5), or the parent plasmid pEGFP-C3 (GFP) (control [ctl]) were transfected into Neuro-2A cells (500 ng DNA/well). (A) At 5 h posttransfection, cells were fixed and stained for membranes with wheat germ agglutinin (WGA). Controls included EGFP-Rab5, which localizes within recycling endosomes (56), and EGFP-C3 which yields a cytosol-localized EGFP. Representative fluorescence staining is shown: EGFP-C3 was present in the cytosol and nucleus, EGFP-Rab5 localized to the perinuclear region, EGFP-LC/A1 localized on the cell membrane, and EGFP-LC/A3 was present in the cytosol, but not localized within the nucleus. (B) Percentage of EGFP-LCA1 or EGFP-LC/A3 colocalized with WGA on the cell membrane (left panel) or percent of EGFP-LCA1 or EGFP-LC/A3 present in the cytosol (right panel) was quantified. Ten random fields of EGFP-transfected cells were scored positive for membrane localization when EGFP colocalized with WGA on the cell membrane or positive for cytosolic localization when EGFP was detected in the cytosol. The percent positive for membrane localization or cytosol localization was scored as the percent EGFP localized/(total number of EGFP-positive cells scored × 100).

Since distinct intracellular localization has previously been shown to correlate with duration of action of the long-lived BoNT/A1 and the short-lived BoNT/E1 LCs (8–10), enhanced green fluorescent protein (EGFP)-LC/A1 and EGFP-LCA3 localization in Neuro-2A cells was examined. EGFP-LC/A1 colocalized with wheat germ agglutinin on the cell membrane, whereas EGFP-LC/A3 was primarily located within the cytosol (Fig. 6). The overall percentage of transfected cells and the fluorescence intensity of the GFP reporter proteins were similar, suggesting that LC/A1 and LC/A3 had similar (short-term) stability.

## DISCUSSION

The molecular mechanism underlying the long duration of BoNTs is an unsolved question in BoNT research. While desirable for therapeutic purposes, long duration of BoNT action after neuronal cell entry, at which point the toxins are not accessible to antitoxin treatments, is also largely responsible for the severity of botulism pathology. We have previously reported that BoNT/A3 has a significantly shorter duration of action than BoNT/A1, -2, -4, and -5 (3, 4). In this study, the molecular basis for the observed unique longevities and pathologies of BoNT/A1 and BoNT/A3 were characterized to determine whether the individual LC or HC domains were rate-limiting steps in BoNT intoxication or whether combinations of LC and HC functions contributed to BoNT intoxication. Analysis of *Clostridium*-produced, recombinant, hybrid toxins, BoNT/A1A3 and BoNT/A3A1, relative to the parent toxins showed that both LC and HC contributed to the specific activity in mice and efficiency of SNAP-25 cleavage in cultured cells (Fig. 2 and 3), while LC defined the duration of BoNT/A action in cultured cells (Fig. 4) and in two mouse models of botulism (Fig. 5). Thus, BoNT potency was a property of both LC and HC, while BoNT duration of action was a property of LC function. These data indicate the feasibility of development of new BoNT therapeutics through the use of subtype chimeras with distinct phenotypes for customization of BoNT duration, without interfering with the sensitivity to antitoxins and therapies (31).

The duration of action of various BoNT serotypes range from a few days to several months. For example, BoNT/E1 has a shorter duration of action than BoNT/A3, resulting in recovery of intoxicated primary rodent neurons within 1 month versus 4 to 5 months for BoNT/A3, and recovery of locally injected mice within 3 to 5 days versus 6 or 7 days for BoNT/A3 (4, 32, 33). In this study, mice locally injected with the BoNT/A3A1 hybrid suffered little overall motor neuron deficiency as tested by rotarod analysis (Fig. 5A), whereas local paralysis assessed by the DAS assay was significant, although of shorter duration than that caused by BoNT/A1A3 (Fig. 5B). This agrees with data derived from long-term activity in cultured neurons (Fig. 4), where BoNT/A3 had some (slowly waning) activity remaining for about 4 months. In contrast, BoNT/E1 has been shown to lead to full recovery of intoxicated neurons after about 1 month (4, 32). The minimal effect of intramuscular BoNT/A3A1 injections on rotarod performance is consistent with previous observations for BoNT/A3 (3) and likely reflects the less severe local paralysis combined with fast recovery of systemic symptoms, which are due to toxin diffusion away from the injection site. The rotarod measures overall motor neuron deficiency of the entire animal versus the local paralysis measured by DAS.

Previous studies on intracellular localization of long-lived LC/A1 and short-lived LC/E1 showed that LC/A1 localized to the plasma membrane of neuronal cell lines, while LC/E1 localized to the cytoplasm (8–10). Examination of the intracellular localization of BoNT/A3 LC-GFP and BoNT/A1 LC-GFP fusion proteins in Neuro-2A cells showed that the distribution of the BoNT/A3 LC-GFP fusion protein in the cytosol was similar to the previously reported distribution for the BoNT/E1 LC-GFP fusion protein, while the BoNT/A1 LC-GFP fusion protein localized to the plasma membrane (Fig. 6). These data are consistent with the hypothesis that intracellular localization of the LC plays a role in determining its half-life inside neuronal cells. However, since the LCs of both BoNT/E1 and BoNT/A3 localize to the cytoplasm in neuronal cells, yet BoNT/E1 has an even shorter duration of action than BoNT/A3, intracellular localization alone is unlikely to determine the intracellular half-life of the various BoNT-LCs and functional recovery. Furthermore, no mechanism directly relating membrane localization and long duration of action has been elucidated. Current studies are addressing the role of the distribution of charged and aliphatic amino acids within the LPH region as contributing to the short half-life of LC/A3 (Fig. 1). Since both N-terminal and C-terminal regions of LC have been implicated in LC membrane localization (10), the LPH region may contribute to localization, but it is not anticipated to be sufficient for intracellular LC targeting to the membrane. Other factors that have been suggested to affect BoNT LC stability inside the neuronal cell include ubiquitination and deubiquitination, tyrosine phosphorylation, a dileucine motif, and a threonine (T420) in the C-terminal portion of the LC, which is conserved between subtypes A1 and A3 (6, 7, 34–36). Future experiments will be needed to determine whether there is a common underlying molecular mechanism, such as intracellular localization, or multiple mechanisms that can determine BoNT LC persistence in neuronal cells.

This study did not investigate the potential role of neurite sprouting in the shorter duration of action of BoNT/A3. Neurite sprouting has been shown in elegant studies to play a role in functional recovery of local paralysis after BoNT/A1 treatment in mice (15, 16). In a recent study, treatment of BoNT-affected NMJs with black spider venom, which destroys the intoxicated synapse, followed by rapid formation of new synapses, has been shown to lead to abrupt and complete recovery from BoNT-induced paralysis (37). These data are consistent with neurite sprouting playing a prominent role in functional recovery of the NMJ after BoNT intoxication in mice. Future studies to elucidate the mechanisms underlying BoNT-LC persistence should consider all aspects that may be involved, including the role of intraneuronal BoNT LC structure, localization, modifications inside the neuronal cell, and cellular responses.

While the current study assessed the molecular organization within BoNT/A subtypes by using recombinant hybrids derived from two different BoNT/A subtypes, several natural BoNT hybrids have been described. Natural hybrids combining functional domains of various serotypes include BoNT/FA, a natural hybrid of BoNT/F and BoNT/A (38, 39) and hybrids of BoNT/C and BoNT/D (1, 40–42). In addition, BoNT/F5 has been described as a natural subtype hybrid consisting of a unique LC and F2-like HC (43). Similarly, BoNT/A3 appears to be a unique class hybrid with a recombination event within an individual serotype (25). Characterization of natural variants within BoNT subtypes may also provide insight into the actions of this family of neurotoxins and may enhance our understanding of neuron biology, such as other regions of LPH within the HC_C_ of BoNT/E currently under investigation.

The current study assessed the molecular organization within BoNT/A subtypes, using recombinant hybrid toxins of BoNT/A1 and BoNT/A3, where BoNT/A3 itself appears to be a natural hybrid with a previously unrecognized region of LPH in the LC. Continued characterization of recombinant and natural variants within BoNT serotypes and subtypes will provide insight into the actions of this family of neurotoxins, enhance our understanding of neuron biology, and generate the next generation of BoNT-based human therapeutics.

## MATERIALS AND METHODS

### Biosafety and biosecurity.

The Johnson laboratory and personnel are registered with the Federal Select Agent Program for research involving botulinum neurotoxins (BoNTs) and BoNT-producing strains of clostridia. The research program, procedures, documentation, security, and facilities are monitored by the University of Wisconsin—Madison Biosecurity Task Force, the University of Wisconsin—Madison Office of Biological Safety, the University of Wisconsin Select Agent Program, and the Centers for Disease Control and Prevention (CDC) as part of the University of Wisconsin—Madison Select Agent Program. Personnel have undergone suitability assessments and completed rigorous and continuing biosafety training, including biosafety level 3 (BSL3) or BSL2 and select agent practices, before participating in laboratory studies involving BoNTs and neurotoxigenic clostridia. All botulinum toxins used in this study are neutralized by the heptavalent botulinum antitoxin hBAT. The creation of the hybrid toxins is considered a restricted experiment according to select agent regulations, and the Johnson laboratory was granted permission to create them. Animal experiments have been approved by the University of Wisconsin IACUC. The Barbieri laboratory and personnel work with exempt amounts of BoNT (42 CFR §§ 73.3, 73.4; 9 CFR §§ 121.3, 121.4; 7 CFR § 331.3) and studies are conducted with BSL1 and BSL2 practices.

### Plasmid construction.

Individual light chain (LC) and heavy chain (HC) gene regions encoding botulinum toxin serotype subtype A1 (BoNT/A1) (GenBank accession number AF461540) and BoNT/A3 (GenBank accession number GQ241940) were amplified by PCR using total genomic DNA isolated from *C. botulinum* strains Hall A-hyper and CDC40234 as the templates, respectively. An approximately 50-bp region located between the LC and HC is homologous between the genes encoding BoNT/A1 and BoNT/A3, and therefore, PCR primers were designed to contain this region in all LC and HC gene fragments. This enabled generation of a seamless junction between the two different subtype gene regions encoding the hybrid BoNT genes *bont*/A1A3 and *bont*/A3A1, using overlap PCR, resulting in hybrid BoNT genes that do not encode additional amino acid residues or protein tags (Table 1). After verification of nucleotide sequences, both recombinant genes were then inserted into clostridial expression vectors pMTL82152 and pMTL83152 (44). The expression constructs were transferred to the nontoxigenic *C. botulinum* expression host strain Hall A-hyper/tox^−^ by conjugation from an *Escherichia coli* CA434 donor strain as previously described (45).

**TABLE 1:** Schematic presentation of recombinant hybrid serotype A BoNTs composed of combinations of subtype A1 and A3 light and heavy chains^a^

Hybrid toxin	Composition	LC gene portion	HC gene portion
rA1A3	LC/A1-HC/A3	1 to 1332 bp of BoNT/A1	1321 to 3879 bp of BoNT/A3
rA3A1	LC/A3-HC/A1	1 to 1320 bp of BoNT/A3	1333 to 3891 bp of BoNT/A1

a LC, light chain; HC, heavy chain; rA1A3, recombinant A1A3 hybrid toxin.

### Botulinum neurotoxins.

BoNT/A1 and BoNT/A3 were purified from *C. botulinum* strains Hall A-hyper and strain CDC40234 (kindly provided by Susan Maslanka and Brian Raphael, Centers for Disease Control and Prevention), as previously described (30, 46). Recombinant BoNT/A1A3 and BoNT/A3A1 were produced in a nontoxigenic strain of *C. botulinum* (Hall A-hyper/tox−) and purified as previously described (45, 47). Briefly, the purification method was similar to that used in BoNT/A1 purification (46) with the following modifications. The ammonium sulfate-precipitated toxin complex obtained from the DEAE chromatography at pH 5.5 was collected by centrifugation and resuspended in 20 mM NaPO_4_ buffer (pH 6.1). The sample was applied to a *p*-aminobenzyl-1-thio-β-d-galactopyranoside agarose affinity column (pABTG agarose) that tightly binds the hemagglutinin of the toxin complex. Toxins were eluted from the column by the addition of 20 mM NaPO_4_ buffer (pH 8.0) containing 0.3 M NaCl (48). BoNT/A1A3 recovered from the pABTG chromatography was further purified on an SP Sephadex column equilibrated with 0.02 M NaPO4 buffer (pH 7.0). The bound toxin was eluted with equilibrating buffer containing 0.3 M NaCl. Toxin purity was confirmed by spectroscopy and SDS-PAGE analyses. The purified toxins were stored in phosphate-buffered saline with 40% glycerol at −20°C until use.

### Cell-based toxicity assay.

Cell-based assays were performed essentially as previously described (49). Human induced pluripotent stem cell (hiPSC)-derived neurons (iCell neurons) were purchased from Cellular Dynamics International (CDI) (Madison, WI) and were seeded into 96-well TPP plates (Techno Plastic Products, Midwest Scientific, Valley Park, MO). The wells had been coated with 0.01% poly-l-ornithine and 8.3 µg/cm^2^ Matrigel (BD Biosciences, East Rutherford, NJ) and were seeded at a density of 35,000 to 40,000 cells per well. Cells were maintained in the provided culture medium according to the company instructions. At 5 to 7 days after the cells were seeding, they were used in the toxin activity and neutralization assays. Primary rat spinal cord (RSC) cells were prepared from embryonic day 15 (E15) Sprague-Dawley rat pups and seeded into 96-well TPP plates. The wells had been coated with 0.01% poly-l-ornithine and 8.3 µg/cm^2^ Matrigel at a density of 50,000 cells/well. RSC cells were maintained in neurobasal medium supplemented with vitamin B27, GlutaMAX, and penicillin-streptomycin (all from Life Technologies) as previously described (50, 51) and used for the BoNT assay after 19 days in culture.

For the BoNT activity assay, serial dilutions of purified BoNTs were prepared in iCell neuron culture medium and added (50 µl) to the hiPSC-derived neurons. Cells were incubated for 48 h at 37°C in 5% CO_2_. The culture medium was removed, and cell lysates were prepared in lithium dodecyl sulfate (LDS) sample buffer (50 µl, Life Technologies).

For the duration of action assay in primary rat spinal cord cells, the RSC cells were exposed to the minimal concentration of each toxin (determined empirically) required to achieve nearly 100% SNAP-25 cleavage. For BoNT/A1A3, 9 U/50 µl culture medium (40 pM) was added to each well, and for BoNT/A3A1, 6 U/50 µl culture medium (10 pM) was added. After 48 h, the culture medium containing the toxin was removed, and cells were washed three times with 300 µl of fresh culture medium to remove residual extracellular toxin. The cells were returned to the incubator and incubated for up to 9 months, with twice weekly replacements of half of the medium. One week after toxin exposure and at monthly intervals, replicates of at least three wells were harvested by cell lysis in LDS sample buffer (75 µl; Life Technologies).

Cell lysates were analyzed by Western blotting for SNAP-25 cleavage as previously described (50, 51). Images were obtained using PhosphaGLO reagent (KPL, Gaithersburg, MD) and a Fotodyne/FOTO/Analyst FX imaging system (Harland, WI), and the cleaved and uncleaved SNAP-25 bands were quantitated by densitometry using TotalLab Quant software (Fotodyne, Harland, WI). Fifty percent effective concentration (EC_50_) values for SNAP-25 cleavage were estimated using GraphPad Prism 6 software and a nonlinear regression with variable slope and four parameters.

### Mouse assays.

Activities of the subtype and hybrid toxin preparations were determined using a standard intraperitoneal mouse bioassay (MBA) as previously described (52, 53). The half-lethal dose of each toxin was defined as 1 mouse 50% lethality dose (LD_50_) unit. *In vivo* duration of action was determined by the digital abduction score (DAS) and rotarod analysis as previously described (3). Briefly, groups of five female ICR mice (Harlan) were injected into the right gastrocnemius muscle with the indicated sublethal amounts of BoNT in 10 µl of GelPhos buffer (30 mM sodium phosphate [pH 6.3] and 0.2% gelatin) using an insulin syringe. The ability of mice to remain running on a rotarod (Med Associates) using an accelerating cycle of 4 to 40 rpm over 5 min was determined daily until full recovery.

### Expression of GFP-LC/A1 and GFP-LC/A3 in Neuro-2A cells.

Enzymatically inactive LC/A1^rym^(1-448, C429S) and LCA3^rym^(1-448, C425S) were engineered as N-terminal green fluorescent protein (GFP) fusion proteins within pEGFP-C3 (10) and shown as GFP-LC/A1 and GFP-LC/A3, respectively (the rym superscript indicates two point mutations within the LC [R363A and Y366F], which reduce catalysis to baseline in a standard SNARE protein cleavage assay [54], while the C425S mutation was engineered to reduce the possibility of heterologous disulfide bond formation). Neuro-2A cells (ATCC CCL-131) were seeded in 24-well plates, and when they reached ~70% confluence, they were transfected with 500 ng of indicated plasmids using Lipofectamine LTX (Invitrogen). At ~5 h posttransfection, cells were fixed and incubated with Alexa Fluor 647-labeled wheat germ agglutinin (WGA^647^) for 1 h at room temperature. Controls included EGFPC1-Rab5 (55) which localizes within recycling endosomes (56), and pEGFP-C3 which yields a cytosol-localized enhanced green fluorescent protein (EGFP). Ten random fields for each transfection were scored as positive for membrane localization when EGFP colocalized with WGA on the cell membrane and/or positive for cytosolic localization when EGFP was detected in the cytosol. The percent positive was scored as percent EGFP localized/(total number of EGFP-positive cells scored × 100).
